# Age-Related Transcriptomic Changes in the Vermiform Appendix

**DOI:** 10.3390/ijms262311399

**Published:** 2025-11-25

**Authors:** Damir Quien, Jelena Korac-Prlic, Katarina Vilović, Zenon Pogorelić, Matija Boric, Ognjen Barcot, Marina Degoricija

**Affiliations:** 1 Department of Surgery, University Hospital of Split, Spinčićeva ulica 1, 21000 Split, Croatia; damir.quien@gmail.com (D.Q.);; 2 Department of Surgery, School of Medicine, University of Split, Šoltanska ulica 2a, 21000 Split, Croatia; 3 Laboratory for Cancer Research, Department of Immunology and Medical Genetics, School of Medicine, University of Split, Šoltanska ulica 2a, 21000 Split, Croatia; 4 Department of Pathology, Forensic Medicine and Cytology, University Hospital of Split, Spinčićeva ulica 1, 21000 Split, Croatia; 5 Department of Anatomy, School of Medicine, University of Split, Šoltanska ulica 2a, 21000 Split, Croatia; 6 Department of Pediatric Surgery, University Hospital of Split, Spinčićeva ulica 1, 21000 Split, Croatia; 7 Department of Medical Chemistry and Biochemistry, School of Medicine, University of Split, Šoltanska ulica 2a, 21000 Split, Croatia

**Keywords:** appendix, aging, transcriptomics, intestinal barrier, oxidative stress, inflammaging

## Abstract

Aging of the gut involves progressive changes in structure, function, and microbial composition, which impact overall health. The vermiform appendix extends from the apex of the cecum; it contains gut-associated lymphoid tissue and serves as a reservoir of gut microbiota. This study investigates histologic and gene expression changes in 20 morphologically normal appendiceal samples obtained from pediatric (*n* = 5), adult (*n* = 8), and geriatric (*n* = 7) patients. Histologic analysis revealed a higher prevalence of lymphoid follicles reduction and the presence of fibrous obliteration of the appendiceal tip in aged samples. RNA sequencing identified 1004 differentially expressed genes (385 upregulated and 619 downregulated; *p* < 0.05) between the adult and geriatric population. Upregulated pathways were enriched for oxidative stress response, cholesterol metabolism, and mucosal barrier maintenance, including NRF2 targets (NQO1, MGST1), suggesting enhanced antioxidant activity. Downregulated genes were associated with synaptic signaling, ion channel regulation, and neuronal adhesion (e.g., GRIA2, RET, NOS1, NCAM2, CNTN1), reflecting age-related decline in enteric neuronal integrity. Across all age groups, 25 protein-coding genes showed progressive expression shifts with aging, including upregulation of CLDN2, MUC2, and GDF15, and downregulation of NOG and NELL2, indicating barrier loosening, chronic inflammation, and reduced regenerative potential. These findings suggest that aging of the vermiform appendix recapitulates key processes of intestinal aging, including oxidative stress, inflammaging, and neuronal loss, supporting its potential use as a model tissue for studying gut aging mechanisms.

## 1. Introduction

The human appendix is a tube-like structure that extends from the apex of the cecum, a part of the large bowel. Also known as the vermiform appendix due to its worm-like appearance, it is present in humans and primates, with few other mammalian species having a similar structure [1]. First described in the 16th century, its function in humans remained unknown, and it was considered a developmental remnant for a long time. A study on primates from the 1980s established that the appendix evolved progressively throughout the species [2]. Histological analyses showed an abundance of lymphoid tissue in the appendiceal mucosa, suggesting an immunological function distinct from the rest of the gut, particularly in the interaction and handling of gut bacteria [3,4]. The presence of microbiota biofilm and the very structure of this organ function as a “safe house” for commensal flora in situations where inoculation is needed after an event that depletes intestinal flora [4]. Currently, the appendix is considered a secondary lymphoid organ that functions as part of the gut-associated lymphoid tissue (GALT). Interesting findings show that in mice, cecal patch removal results in diminished IgA diversity in the colon [5]. From a clinical perspective, appendectomy is not associated with long-term adverse effects; however, it can be associated with a reduced risk of developing ulcerative colitis later in life [6,7,8].

Aging represents a complex process of physical and mental decline, characterized by cellular and organ dysfunction, accumulation of DNA damage, and resulting age-related changes in gene expression. Central to systemic aging, changes in the gut can significantly affect one’s overall health. The aging intestine undergoes a series of morphological and functional changes that can impair its function and tissue homeostasis [9]. Most important are the changes occurring in intestinal stem cells (ISCs), the enteric nervous system, and the intestinal epithelium, which are intricately connected to gut microbiota [10]. Functional and structural alterations in the intestinal mucosa during aging disrupt the mucosal barrier between the host and the microbiota. ISCs play a central role in the regeneration of intestinal epithelium throughout an individual’s life, allowing for the proper functioning and structural integrity of the gastrointestinal tract [11]. Downregulation of the essential transcription factors (*Egr1*, *Irf1*, and *FosB*) in aging mice, caused by epigenetic alterations, reduces the regenerative capacity of ISCs, mainly due to a decline in canonical *Wnt* signaling [12]. In vivo studies in animal models show impaired DNA damage response function in ISC, leading to cellular dysfunction and tumorigenesis in older mice [13]. The intestinal mucosal barrier selectively enables the uptake of nutrients and water while preventing the entry of pathogens and toxins into the organism [14]. Tight junctions form a continuous seal between epithelial cells; they are composed of protein complexes, including occludins and claudins, which regulate paracellular permeability [15,16]. Deregulation of paracellular permeability results in “leaky gut”, which is in tight relation with microbiota dysbiosis. The gut microbiome in older adults shows a reduction in beneficial commensals, which can contribute to inflammaging through the leakage of proinflammatory cytokines and, eventually, to carcinogenesis [17]. Aging is also closely related to oxidative stress and neuronal vulnerability through age-dependent decline in antioxidative defenses, which result in the impaired ability of tissue to neutralize reactive oxygen species (ROS) [18]. Concomitant mitochondrial disfunction increases the generation of ROS, promoting the oxidative damage to neuronal membranes and DNA [19]. In addition to this, chronic low-grade inflammation, due to reduced paracellular permeability, further amplifies oxidative stress and immune dysregulation [20].

Recent transcriptomic studies have provided several important insights into specific age-related processes in the gut. The scRNA-seq atlas of the healthy human gut, covering fetal, pediatric, and adult stages across multiple anatomical regions, found significant shifts in cell lineage proportions across life stages, as well as regional variation (small intestine vs. colon) in gene expression signatures and cell-type composition [21]. It was demonstrated that aging substantially increases core fucosylation of the apical surface of intestinal epithelial cells. The study by Drokhlyansky et al. established the first transcriptomic blueprint of the mouse and adult human enteric nervous system (ENS), identifying neuronal and glial subtypes, as well as conserved and species-specific features that are also location- and age-specific [22]. Transcriptomic studies in mice demonstrated that aged ISCs with impaired regenerative capacity contribute to inflammaging by upregulating MHC-II antigen presentation, whereas a single-cell RNA study of ISCs in mice showed suppression of the Lgr5 stem cell marker in aged stem cell clusters [23,24]. RNA sequencing of the mouse aged colon and mesenteric lymph nodes revealed that gut barrier integrity in aged mice can be ameliorated by microbiota depletion [25]. Finally, a very recent scRNA-seq study on fetal-to-preschool-age appendices established a comprehensive cellular atlas that describes 76 distinct cell types in the developing human appendix and revealed that the BMP signaling pathway plays a crucial role in the development of appendiceal epithelial cells [26].

Transcriptomic studies on gut aging are limited, and the mechanism of inflammaging in human tissues remains a complex, incompletely understood process. This is the first study to characterize both histologic and transcriptomic alterations in the healthy human vermiform appendix across the full age spectrum, from pediatric to geriatric individuals. Our work highlights histologic changes including fibrous obliteration of the appendiceal tip and a marked reduction of lymphoid follicles in vermiform appendices in the context of aging. We identify age-related transcriptomic signatures implicated in oxidative stress, inflammaging, and neuronal loss, providing novel insight into how aging remodels appendix biology at both the tissue and gene-expression levels. Understanding the intricate mechanisms of aging that lead to tissue dysfunction may hold the key to better treatment of age-related gastrointestinal diseases in the future.

## 2. Results

### 2.1. Baseline Patient Characteristics

**Figure 1:**
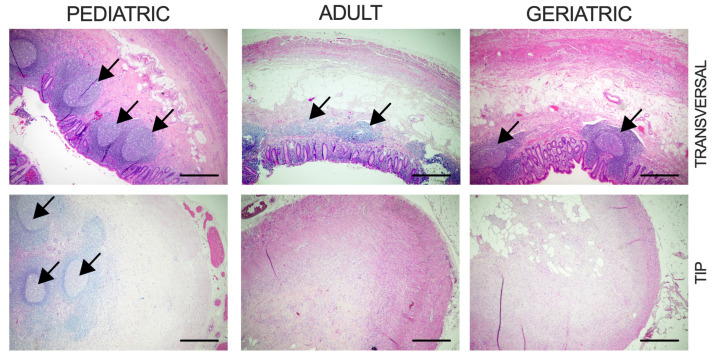
Histological view of representative appendiceal samples in distinct age groups. Images show the transverse section of the lumen and longitudinal section of the appendiceal tip. Arrows denote lymphoid follicles. Scale bar represents 500 μm.

In this study, we analyzed samples of normal vermiform appendices from individuals in different age groups. We included five pediatric samples (from individuals aged 18 years and younger), eight adult samples, and seven geriatric samples (from individuals aged 65 years and older). The mean age of the geriatric group was 74.6 ± 8.5 years, compared to a mean age of 39.5 ± 10.6 years in the adult and 16 ± 2.1 years in pediatric group. The average body mass index (BMI) was comparable between geriatric (25.8 ± 4.7 kg/m^2^) and adult patients (26.0 ± 5.4 kg/m^2^) and significantly lower in the pediatric group (18.8 ± 3.0 kg/m^2^). Reduction of lymphoid follicles and fibrous obliteration of the tip of the vermiform appendix were observed at a higher frequency in the adult and geriatric population. Fibrous obliteration of the tip of the appendix was present in 20% of pediatric samples in comparison to 75% prevalence in the adult population and 71.4% in the geriatric population. Overall prevalence of fibrous obliteration of appendiceal tip in our samples was 65%. The pediatric population consisted solely of female patients due to the unavailability of male samples (Figure 1 and Table 1).

### 2.2. Transcriptomic Alterations in the Appendix of the Aged Population

**Figure 2:**
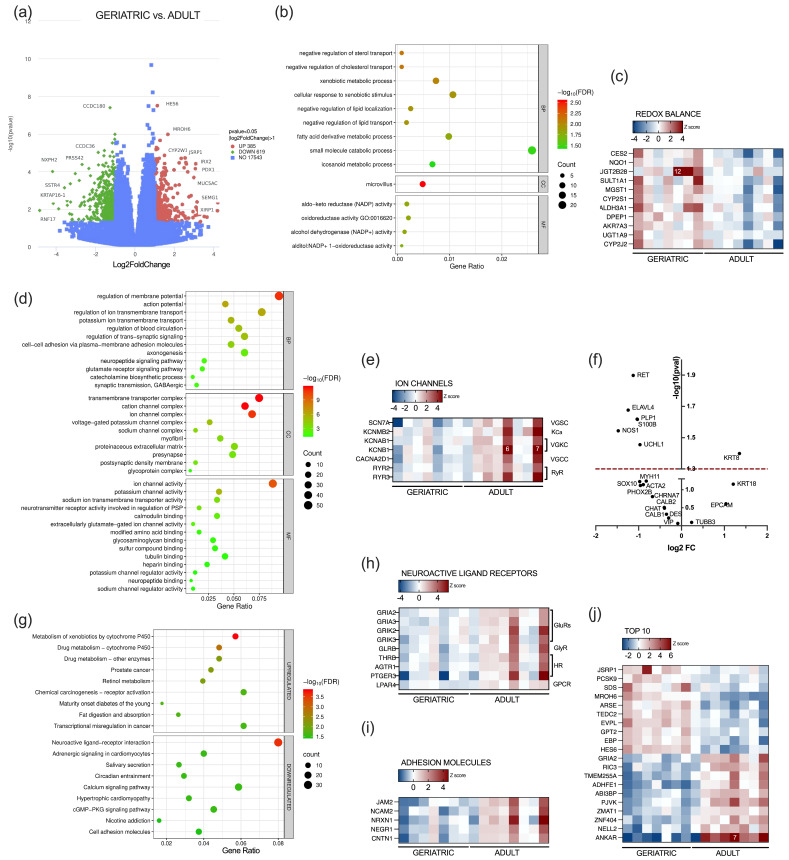
Transcriptomic alterations in the appendix of the aged population. (**a**) Volcano plot illustrating DEGs between geriatric and adult population; (**b**) GO enrichment bubble plot showing upregulation of specific gene signatures between geriatric and adult population for GO category Biological Processes (BPs); cellular compartment (CC) and molecular function (MF); (**c**) heatmap of specific genes related to drug detoxification, oxidative stress protection, and gut homeostasis upregulated in the geriatric population; (**d**) GO enrichment analysis showing downregulation of specific BPs in the geriatric population; (**e**) heatmap depicting specific ion channel genes downregulated in the geriatric population; (**f**) volcano plot for specific gene markers of neuronal, epithelial, and muscle cells; (**g**) KEGG pathway analysis of upregulated and downregulated processes in geriatric population; (**h**) heatmap illustrating the decreased expression of specific gene sets in the neuroactive ligand–receptor interactions pathway; (**i**) heatmap of neuroactive ligand receptors; (**j**) heatmap demonstrating the top 10 most significantly upregulated and downregulated DEGs in the geriatric population (FDR < 0.01). Numbers on heatmaps denote values out of legend range.

To investigate transcriptomic changes associated with aging in the appendix, we analyzed healthy appendiceal tissue from seven geriatric and eight adult patients. RNA sequencing of cross-sections from the vermiform appendix revealed extensive transcriptomic alterations associated with advanced tissue aging. Specifically, we identified 385 protein-coding genes that were significantly upregulated by more than 2-fold and 619 genes that were significantly downregulated, with *p*-values < 0.05 (Figure 2a).

We performed Gene Set Enrichment Analysis (GSEA) for functional characterization. Gene Ontology (GO) enrichment analysis revealed upregulation of biological processes related to the apical membrane of enterocytes and cholesterol metabolism (Figure 2b). Furthermore, processes associated with drug detoxification, oxidative stress protection, and gut homeostasis—including NRF2 target genes, among which are oxidoreductases NQO1 and microsomal glutathione peroxidase MGST1—were also significantly upregulated (Figure 2b,c).

Conversely, significant downregulation was observed in biological processes involving the regulation of membrane potential and synaptic transmission. Several ion channels, including subunits of voltage-gated sodium, potassium, calcium, and ryanodine receptors, were significantly downregulated (Figure 2d,e). Since age-related decline in enteric neurons and gut neuromuscular function is well documented, we specifically investigated the expression of several common ENS neuronal markers, as well as controls for tissue composition. We observed significant downregulation of RET, a marker of ENS progenitors, pan-neuronal markers ELAVL4 and UCHL, NOS1, as well as glial markers S100B and PLP1 (Figure 2f). KEGG pathway analysis further revealed reduced neuroactive ligand–receptor interactions, attributed to decreased expression of glutamate receptor subunits (GRIA2, GRIA3, GRIK2, and GRIK3) and glycine receptor subunit GLRB. Several hormone receptors, such as thyroid hormone receptor THRB, angiotensin II receptor AGTR1, lysophosphatidic acid receptor LPAR4, and prostaglandin E receptor PTGER3, were also significantly downregulated (Figure 2g,h). In addition to this, the downregulation was observed for important cell adhesion molecules—including tight junction protein JAM2 and neuronal adhesion molecules NCAM2, NRXN1, NEGR1, and CNTN1 (Figure 2i).

The most substantial evidence for differential upregulation (FDR < 0.01) was observed for genes necessary for metabolic reprogramming, including regulators of cholesterol metabolism, PCSK9, and EBP, as well as amino acid convertases SDS and GPT2. In addition, several tissue maintenance genes (EVPL2, ARSE, and TEDC2) and muscle remodeling genes (JSRP1 and HES6) were consistently upregulated in the geriatric population. The strongest downregulation was detected for genes involved in synaptic function (GRIA2, NELL2, and RIC3), the metabolic metalloenzyme ADHFE1, and the structural proteins ABI3BP, ANKAR, and TMEM255A. We detected low overall expression of genes ZMAT1, ZNF404, and PJVK (Figure 2j).

### 2.3. Sex-Specific Transcriptomic Alterations in Vermiform Appendix

**Figure 3:**
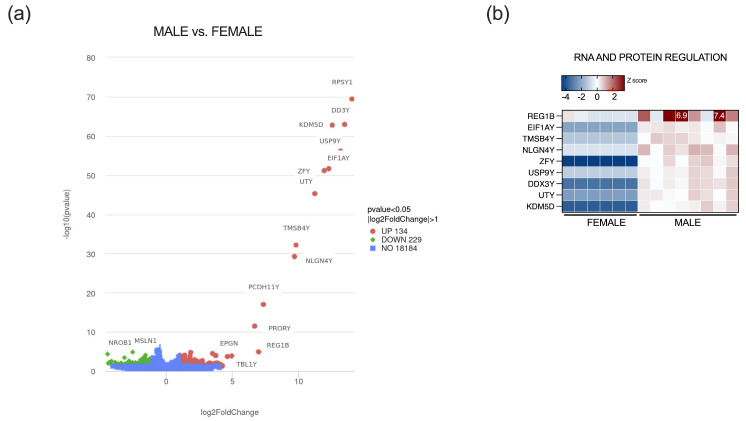
Sex-specific transcriptomic alterations in the appendix: (**a**) volcano plot illustrating sex-specific DEGs in male and female population; (**b**) heatmap showing nine significantly upregulated genes in male population (FDR < 0.05). Numbers on heatmaps denote values out of legend range.

We next investigated the adult and geriatric sample population for sex-specific transcriptomic alterations to identify 363 differentially expressed genes, 134 upregulated and 229 downregulated genes in males with a 2-fold change and *p*-value < 0.05 (Figure 3a). After stringent filtering for lowly expressed genes, only nine genes were differentially expressed between male and female samples, with an FDR < 0.05. Most prominently upregulated genes in males were expectedly major Y chromosome genes, such as ribosomal protein RPS4Y1, RNA helicase DDX3Y, male-specific histone demethylase KDM5D, ubiquitin-specific protease USP9Y, translation initiation factor EIF1AY, transcription factor ZFY, protocadherin PCDH11Y, and NLGN4Y, among others (Figure 3b). Due to a low number of DEGs, functional analysis could not be reliably performed.

### 2.4. Progressive Age-Dependent Differential Expression of Genes in the Appendix

Gene expression in the vermiform appendix of the pediatric population was compared to expression in adult and geriatric individuals to identify genes whose expression levels consistently change with aging but are not sex-specific. Principal component analysis (PCA) of appendix transcriptomes revealed age-dependent clustering of samples. Pediatric samples grouped tightly around the PCA origin, whereas adult samples clustered more compactly, but with 37.6% variance along PC1. Geriatric samples displayed the greatest dispersion (14.6% variance along PC2) (Supplemental Figure S1).

**Figure 4:**
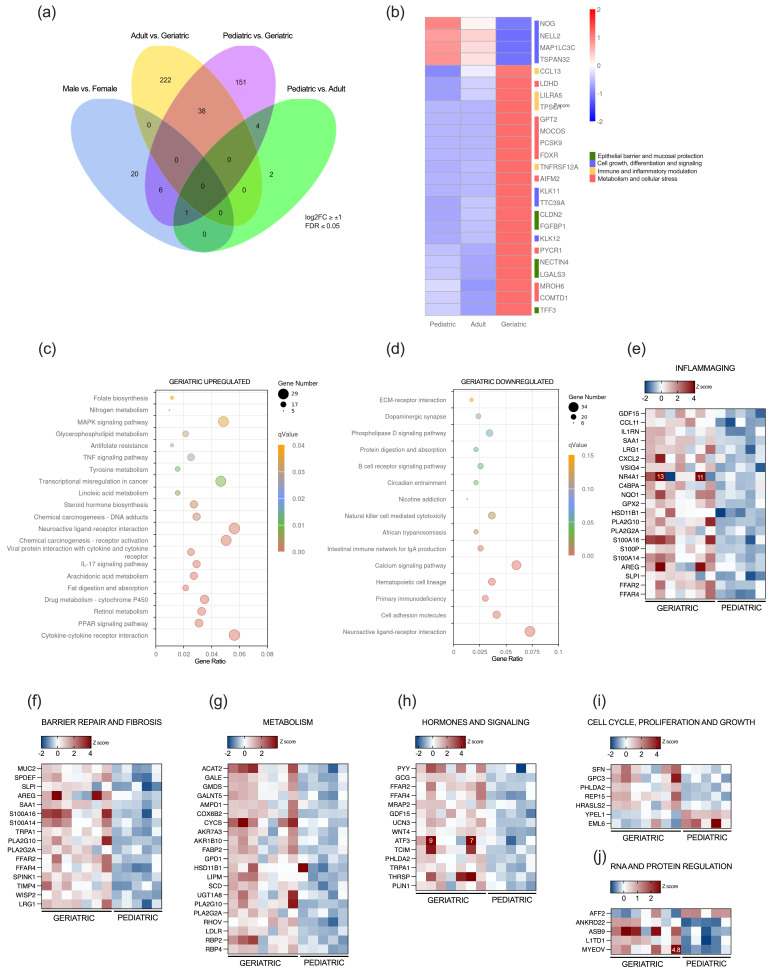
Progressive Age-dependent Differential Expression of Genes in the Appendix: (**a**) Venn diagram showing DEGs across all study groups, log2FC ± 1, FDR < 0.05; (**b**) heatmap of differentially expressed genes across all three age groups; (**c**) KEGG pathway analysis showing upregulated pathways in the geriatric compared to pediatric population; (**d**) KEGG pathway analysis illustrating pathways downregulated in the geriatric compared to pediatric population; (**e**–**j**) heatmaps of selected differentially expressed genes between pediatric and geriatric population grouped according to their function (FDR < 0.05). Numbers on heatmaps denote values out of figure legend range.

Thirty-eight DEGs across three age groups were further filtered for low-expression genes, defining a 25-protein-coding gene set, which is differentially regulated during appendiceal aging (Figure 4a,b). Progressive differential expression was detected for several proteins regulating cell growth and differentiation; downregulation was observed for NOG, NELL2, MAP1LC3C, and TSPAN32, whereas kallikrein-related peptidases KLK11 and KLK12, and TTC39A, were upregulated. Several proteins involved in epithelial barrier integrity, including CLDN2, NECTIN4, FGFBP1, and TFF3, were significantly upregulated across age groups, as well as inflammatory modulators such as CCL13, LILRA5, TPSG1, and TNFRSF12A. In addition, several genes involved in cellular metabolism, including alanine aminotransferase GPT2, PCSK9, FDXR, AIFM2, COMTD1, MOCOS, and PYCR1, were also markedly upregulated in the geriatric population (Figure 4b).

Gradual differential gene expression was also analyzed between the pediatric and geriatric populations, excluding sex-associated DEGs and previously explored DEGs in the adult and geriatric groups. Functional analysis of KEGG pathways revealed upregulation of various metabolic and biosynthetic processes, signaling pathways including MAPK, TNF, and IL17, as well as neuronal and endocrine regulatory processes. Similar to the functional differences observed between adult and geriatric appendices, several upregulated KEGG pathways were related to regulation of membrane potential and neurotransmitter levels, lipid metabolic processes, and positive regulation of the inflammatory response and chemokine production (Figure 4c). Downregulation was observed for pathways that regulate both innate and adaptive immune responses, cell adhesion and extracellular matrix receptors, and neuronal and synaptic signaling, among others (Figure 4d).

Stringent filtering (FDR < 0.05, log2FC > ±1) of gradual age-associated DEGs between pediatric and geriatric appendices resulted in 151 differentially expressed protein-coding genes (Table S1). Upregulation was observed for genes involved in inflammaging, mucosal barrier repair and fibrosis, metabolism, hormone and signaling molecules, as well as genes essential for cellular metabolism, hormonal and cellular signaling, and cell cycle, proliferation, and growth. Selected genes are represented in the heatmap panels in Figure 4e–j.

## 3. Discussion

Tissue aging is a progressive process of multicellular structural and functional decline that ultimately leads to tissue atrophy and age-related diseases. Hallmarks of intestinal aging include the accumulation of DNA damage, metabolic alterations, chronic inflammation, and disruption of the mucosal barrier, which is interrelated to gut microbiota dysbiosis. The human vermiform appendix functions as a specialized segment of the intestine, supporting gut immunity and maintaining gut microbiota. As a distinct part of GALT, the appendix contains an abundance of lymphoid follicles, which appear around the second week after birth and are most numerous at puberty. With age, they diminish in number and size, but may persist in old age, which is in line with the histologic findings in our samples [27,28,29].

Fibrous obliteration of the appendix represents a benign finding in the appendix. Studies suggest that the etiology of this finding is consequential to recurrent subclinical inflammation caused by neuroendocrine cell hyperplasia in the submucosa and lamina propria of the appendix, which is subsequently replaced by fibromyxoid stroma [30]. Previous studies describe a 0.8 to 20.5% prevalence of fibrous obliteration after appendectomy, with rates being higher after incidental appendectomy [31,32,33]. Our results on incidental appendectomy samples suggest that fibrous obliteration of the tip of the appendix is frequent, but not significantly more prevalent in geriatric and adult samples in comparison to pediatric samples, which is in line with other studies with a small sample size [34]. However, larger studies of non-inflamed appendix samples should examine the possible relationship between lymphoid follicle reduction and the presence and extent of fibrous obliteration in the context of aging.

Our data demonstrate that specific transcriptomic alterations accompany histologic findings in aged appendices. Transcriptomic findings in adult and geriatric appendices reflect an intricate relationship between increased oxidative damage, loss of neuronal plasticity, and fibrotic remodeling in this segment of the gut. It is known that aging is driven by impaired respiratory chain function and mitochondrial damage, as well as the decline in the activity of antioxidant enzymes [35,36,37]. Chronic low-grade inflammation caused by mucosal barrier deterioration and microbiome dysbiosis further increases the production of reactive oxygen species (ROS) [38]. NRF2 is a master transcriptional regulator of cellular defense against oxidative and metabolic stress. It controls the expression of numerous genes by binding to antioxidant response elements in their promoter regions and consequently upregulates their expression [39,40]. Our findings in the aging appendix indicate activation of NRF2 signaling, as evidenced by the upregulation of several NRF2 target genes, notably NQO1 and MGST1, which have essential cellular antioxidant activity [41]. The role of different NRF2 downstream targets was not extensively studied in the context of the aging human gut; however, it is known that the basal expression of NRF2 target genes in specific tissues increases with age, but inducibility upon stress stimulus declines, because of an age-associated impairment known as NRF2 blunting [42,43,44]. Inducible NFR2 activation is generally considered tissue protective; however, its role in the gut during inflammation may be protective in the short term but potentially pro-tumorigenic in the long term, as demonstrated in the study by Kruse et al., which describes epithelial adaptation in NCM460 cells and apoptosis resistance under ROS challenge from inflammatory myeloid cells [45]. Studies in mice demonstrated that NRF2 activation in enterocytes enhances NQO1 expression and induces gut remodeling, whereas NRF2 pharmacologic blockade improves age-dependent distal colon contractility in female mice [46,47]. Considering the versatility of NRF2 downstream effects, which might be tissue-specific, future studies are needed to clarify the role of increased basal expression of NRF2 targets in context of gut inflammaging.

Strong evidence links aging to a loss of myenteric neuronal density (reviewed in [48]). Changes in the ENS result in gastrointestinal dysfunction and may contribute to age-related pathologies. Progressive loss of enteric neurons and reduction in interstitial cells of Cajal result in gastrointestinal dysfunction manifested through weakened secretion and gut motility [48,49]. In relation to this, we observe downregulation of gene expression for RET tyrosine kinase, which sustains tissue maturation and proliferation of enteric neurons and enteroendocrine cells [50]. Single-cell sequencing of human and mouse ENS revealed distinct expression programs, which depend upon location, circadian phases, and aging [22]. Our results demonstrate downregulation of important ion channel genes that regulate neuronal excitability and calcium homeostasis. Voltage-gated potassium channels are susceptible to oxidative stress and the observed age-related downregulation in KCNAB1, KCNB1, and KCNMB2 and CACNA2D1 in the appendix could compromise regulation of neuronal excitability and contribute to calcium dishomeostasis [51,52]. Altered calcium signaling is closely linked to cellular ROS imbalance; it can lead to an elevation of cytosolic calcium and trigger apoptosis, resulting in neuronal loss, slowed intestinal transit and impaired peristalsis [53,54]. In addition to this, glutamate receptors, critical for excitatory neurotransmission in the human gut, play a major role in neuronal calcium regulation. ENS expresses several types of glutamate receptors, including α-amino-3-hydroxy-5-methyl-4-isoxazolepropionic acid (AMPA) receptors, which mediate fast excitatory synaptic transmission. GRIA2 (GluA2) AMPA receptor subunits are crucial for regulating Ca^2+^ permeation and voltage rectification [55,56,57]. We observed downregulation of the GRIA2 subunit of AMPA receptors in conjunction with its transcriptional regulator ADAR2 (log2FC = 0.8, FDR = 0.07), which suggests a pathological shift in neuronal calcium regulation, which can lead to excitotoxic vulnerability [58]. Additional downregulation of several neuronal adhesion molecules (NCAM2, NRXN1, NEGR1, and CNTN1) coherently suggests a breakdown in synaptic architecture and stability [59,60].

Interestingly, we observed an increase in junctional sarcoplasmic protein that regulates CaV1.1-RyR1 coupling [61]. JSRP1 is primarily a marker of contractile cells; thus, the observed upregulation indicates fibrotic remodeling longitudinally throughout the appendiceal wall, which is evident mainly at the appendiceal tip. Low-grade fibrotic aging, sometimes termed “fibroaging”, is observed in other organs and throughout the gut, where it is associated with gut dysbiosis and inflammation [62,63]. Significant downregulation of extracellular matrix protein ABI3BP is another indicator of increased fibrosis because it promotes differentiation over proliferation, as demonstrated in Abi3bp knock-out mice after myocardial infarction and in dilated cardiomyopathy of human hearts [64,65].

Transcriptomic analysis of sex-specific gene expression changes revealed a relatively small number of significant DEGs, most of which are expectedly located on the Y chromosome and have previously been reported to exhibit a high male-to-female ratio across different human tissues [66]. EIF1AY, DDX3Y, NLGN4Y, UTY, and ZFY genes, which are highly expressed in males, are often used in age-estimation models because their DNA methylation status changes reliably with aging, making them useful biomarkers of biological aging in males [67,68]. One interesting finding is increased expression of REG1B, a gene for a small, secreted protein primarily expressed in epithelial tissues during injury. It has anti-apoptotic and proliferative properties that help repair intestinal lining, but when overexpressed in cancer, it promotes cancer cell proliferation and invasion [69,70].

We observed more specific DEGs between the adult and geriatric population than between the pediatric population compared to the adult and geriatric population, which was also previously described in human brain and epidermal tissue as higher stochasticity in age-related expression changes during aging [71,72,73]. Our data demonstrate that genes that are progressively downregulated or upregulated from pediatric age to adulthood and into geriatric age are associated with key hallmark processes of intestinal aging. We observed downregulation of several vital genes for cell growth and differentiation. Noggin (NOG), a well-described BMP antagonist, is expressed in the intestinal stem cell niche. NOG is necessary for the maintenance of stemness in Lgr5+ ISCs, and the observed downregulation might consequently lead to a reduction in the number of ISCs, as previously described in age-related occurrence in mice [74,75,76,77,78,79]. NELL2 is a secreted glycoprotein enriched in neurons and not extensively studied in the enteric nervous system. Animal studies demonstrate that loss of NELL2 causes reduced neuronal survival and differentiation, impairs synaptic plasticity, and increases vulnerability to neurotoxic stress [80,81]. Increased cellular stress can be further promoted by downregulation of one of the core autophagy proteins, MAP1LC3C, which leads to impaired lysosomal degradation. Finally, tetraspanin 32 (TSPAN32) is also downregulated. TSPAN32 is expressed in GALT-associated T-cells, where it functions as a negative regulator of T-cells; thus, its downregulation might disrupt the balance between tolerance to commensals and immunity to pathogens [82,83].

Disruption of the mucosal barrier represents another crucial feature of intestinal aging. Claudin-2, a pore-forming tight junction protein, creates cation-selective, water-permeable channels in the paracellular space, thereby loosening the epithelial barrier. The expression of CLD2 is a primary determinant of intestinal barrier tightness; by selectively conducting sodium, potassium, and water, CLD2 lowers transepithelial electrical resistance (TER) and enables efficient fluid reabsorption [84,85]. Overexpression of CLD2 causes a decrease in TER and increases the paracellular flux of ions, resulting in a “leaky gut” that contributes to dysbiosis, intestinal inflammation, and ineffective pathogen control [84]. Galectin-3 (LGALS3) is another tissue and serum marker of intestinal inflammaging that was upregulated in the geriatric population. Galectin-3 is a β-galactoside-binding lectin that regulates cell adhesion and extracellular matrix remodeling as well as inflammation and fibrosis. Numerous studies describe the implication of galectin-3 in the pathogenesis of many important human diseases, including heart failure, chronic kidney disease, liver and pulmonary fibrosis, as well as cancer. In the intestine, galectin-3 is predominantly expressed in the differentiated enterocytes of the villous tip [86]. It interacts with mucin-2 (MUC2), and it is essential for host–microbiome homeostasis [87,88,89,90,91]. Downregulation of galectin-3 was demonstrated in inflamed intestinal mucosa of patients with inflammatory bowel disease, but elevated serum levels are present in patients with ulcerative colitis and Crohn’s disease [92,93]. Here we observe upregulation of galectin-3 in non-inflamed appendiceal mucosa of older people, which might represent a compensatory mechanism to loosen the intestinal barrier, since galectin-3 was shown to tether with N-cadherin, occluding, and other tight-junction components to reinforce epithelial seal integrity and promote re-epithelialization [94,95,96,97].

Gradual changes in the aging appendix, evaluated by comparison between pediatric and geriatric samples, further emphasized the key hallmark processes in the aging gut. KEGG pathway analysis revealed upregulation of TNF and IL17 and MAPK signaling pathways in addition to downregulation of phospholipase D signaling, processes directly linked to “inflammaging” and cellular senescence [98,99,100]. Upregulation of several genes, including SPDEF, AREG, REG4, and MUC2, among others, is indicative of active intestinal repair. Intestinal mucus is composed of O-glycosylated MUC2 produced by goblet cells. Our results depict increased activity of goblet cells in the aged appendix, characterized by a significant increase in MUC2 expression in the geriatric population, which is in line with previous findings that show an increase in the number of secretory lineage cells with aging [77]. MUC2 is commonly upregulated in chronic inflammation, and its expression is regulated through the NF-κB transcription factor, which can be activated by LPS, TNF, or cytokines, including IL-4 and IL-13. Studies on MUC2 expression in the colon of aged mice reveal impaired mucus barrier integrity and decreased MUC2 expression; thus, the observed increase in the appendix may be site-specific [101]. This specific increase in MUC2 was associated with elevated co-expression of also goblet-specific kazal-type protease inhibitors SPINK4 and SPINK1, as well as a significant increase in expression of tetraspanin-8, which is a gatekeeper of excessive mucin excretion [102]. Considering also the increased expression of the anti-apoptotic serine-protease inhibitor SLPI, olfactomedin-4 (OLFM4), and the growth factor-like peptide REG4, which promote epithelial proliferation, we observed an age-progressive reparative response to low-grade chronic inflammation in the aged human appendix [77,103]. In addition to this, we detected upregulation of growth and differentiation factor GDF15 and the chemokine CCL11, well-described biomarkers of “inflammaging”. Studies show a very strong positive correlation between GDF15 levels and chronological and biological age in human lifespan, and it is considered an essential component of “senescence-associated secretory phenotype” [104,105,106]. Upregulation of CCL11, a chemokine important for eosinophil recruitment, which was demonstrated to reduce neurogenesis and increase microglial ROS production and excitotoxic neuronal death in the brain, might also contribute to age-related changes in the ENS [107,108].

Our study demonstrates that age-related transcriptomic alterations in the vermiform appendix recapitulate key hallmark processes defined in the context of aging, corroborating the idea that the vermiform appendix could be used as a tissue model for gut aging. We observed an upregulation of genes involved in ROS clearance, biomarkers of “inflammaging” and fibrotic remodeling as well as downregulation of genes crucial for functioning of ENS and maintenance of the intestinal barrier, including neuroactive ligand receptors, ion channels, and adhesion molecules. Preventing oxidative damage in the aging gut requires coordinated support of endogenous antioxidant systems, preservation of mitochondrial function, reinforcement of mucosal barrier integrity, and reduction of chronic low-grade inflammation, all of which collectively limit ROS accumulation and protect aging intestinal tissues [107,108].

This study has several limitations. The sample size is relatively small, and future research involving larger cohorts will be necessary to validate and extend these findings. In addition, the study is primarily descriptive in nature and relies on transcriptomic profiling without experimental validation. As such, further in-depth, mechanistic investigations will be essential to determine the functional relevance of the observed changes. Moreover, aging-related processes in the gastrointestinal tract may vary considerably by anatomical location, and the appendix may not fully represent all regional features of gut aging. Comparative studies across different gut segments will therefore be important to further assess the suitability of the human vermiform appendix as a model for specific age-related mechanisms. Aging promotes diseases; therefore, understanding the intricate mechanisms underlying aging and leading to gut dysfunction may hold the key to a better treatment of age-related gut diseases in the future.

## 4. Materials and Methods

### 4.1. Patient Samples

This cross-sectional study included 20 vermiform appendix tissue samples from patients who had undergone surgical treatment at University Hospital of Split from October 2022 to August 2023. Both children and adults who were candidates for an incidental appendectomy as part of an elective or emergency abdominal procedure were included in the study. Before the surgical procedure, informed consent was obtained from the patient or their legal guardian. Patients suffering from disseminated malignant disease, inflammatory bowel diseases, and patients who received neoadjuvant chemoradiotherapy and immunotherapy were not included in the study. Appendix specimens for RNA isolation were sampled from a full-thickness tissue cross-section slice in the proximal third of the appendix immediately after appendectomy was completed, preserved in RNA*later* reagent (Ambion), and further stored at −80 °C.

The study was approved by the Ethics Committees of the University Hospital of Split (Approval number 500-03/22-01/90; Date of approval: 14 June 2022) and the School of Medicine (Approval number 003-08/22-03/0003; 9 December 2022).

### 4.2. Histological Analysis

The remaining tissue from the appendix specimens was formalin-fixed, paraffin-embedded, and four µm-thick sections were deparaffinized, H&E-stained (Sigma-Aldrich, Darmstadt, Germany), and analyzed independently by two experienced pathologists from the Department of Pathology at the University Hospital of Split. Microscopy images were taken with an Olympus BX43 microscope (Olympus Corporation, Tokyo, Japan). Only morphologically normal appendices were included for further analysis.

### 4.3. RNA Preparation and Sequencing

Appendix samples stored in RNA*later* (Ambion) were thawed on ice. The tissue was disrupted using a Minilys homogenizer (Bertin Technologies, Montigny-le-Bretonneux, France), and total RNA was purified using the standard Trizol protocol (Invitrogen, Carlsbad, CA, USA) and dissolved in DEPC-treated water. The quality of total RNA was assessed using a Nanodrop 2000 (Thermo Fisher Scientific, Waltham, MA, USA) and an Agilent 2100 Bioanalyzer (Agilent Technologies, Santa Clara, CA, USA). Only samples with clear electropherogram profiles and acceptable RIN values were advanced to library preparation. Poly-A tail selection was performed for mRNA enrichment. First-strand cDNA was synthesized with random hexamer primers after fragmentation, followed by the second-strand cDNA synthesis using dTTP for a non-directional library. The cDNA library underwent an additional quality control check to ensure proper fragment size and concentration. The Illumina NovaSeq X Plus platform was used for 40 million reads, with paired-end 150 bp sequencing. An average of 92 million raw reads per sample were filtered for low-quality or reads with adapters. Around 95% of reads were retained as clean reads, and the overall base-calling error rate was 0.01 across all samples. Quality scores were consistently high, with Q20 averaging at 97.4% and Q30 values around 92–93%. HISAT2 version 2.0.5 was used for mapping to the human reference genome GRCh38 with an average unique mapping rate around 88%. Quantification of mapped reads was performed using featureCounts (version 1.5.0-p3), and differential analysis was performed with DeSeq2 (version 1.20.0). Genes with log_2_ fold-change ≥ ±1 (i.e., fold-change ≥ 2) and *p*-value ≤ 0.05 were considered differentially expressed. Genes considered adequately expressed and discussed in the Discussion section have FPKM values greater than 1 in two or more samples and a corrected *p*-value (Benjamini–Hochberg method) below 0.1.

Gene ontology enrichment analysis and KEGG analysis were performed on the NovoMagic cloud platform (https://ocsseurope.novogene.com/oauth/login), accessed on 10 May 2025). Complementary Gene Set Enrichment Analysis (GSEA) was conducted using the GenePattern implementation of GSEA with 1000 permutations per gene set, referencing curated MSigDB collections.

Graphical data presentation was constructed using the NovoMagic cloud platform or the online Bioinformatics platform (https://www.bioinformatics.com.cn, accessed 25 May 2025). GraphPad Prism software (version 10.4) was used to create median-based Z-score heatmaps on FPKM-normalized RNA sequencing data.

### 4.4. Statistical Analysis

Categorical variables are presented as percentages and ratios, and patient age and BMI were given as the mean ± SD and 95 CI (Table 1). The normality of the baseline patient sample characteristics was assessed using the Shapiro–Wilk test. Student’s *t*-tests were performed to test differences between means, and Fisher’s exact test was used to test the dependence of two categorical variables, respectively. Statistical analyses and graphing were performed using GraphPad Prism software (version 10.4, La Jolla, CA, USA).

## 5. Conclusions

In conclusion, aging induces histologic changes concomitant with progressive alterations in the transcriptional profile of the human vermiform appendix. A decline in lymphoid follicle density and fibrous obliteration of appendiceal tip is evident even in pediatric samples, suggesting that the aging-related remodeling of appendiceal immune tissue may start in early life. A transcriptomic signature of progressive aging from pediatric to geriatric age is indicative of barrier weakening, chronic low-grade inflammation, and diminished regenerative capacity. A geriatric transcriptomic profile demonstrates increased antioxidant activity and downregulation of genes involved in synaptic signaling, ion-channel modulation, and neuronal adhesion. Collectively, these findings highlight the changes in gene expression related to mucosal barrier deterioration, an increase in ROS, and impairment in synaptic stability, which lead to inflammaging, thus demonstrating that appendiceal aging recapitulates hallmark processes related to aging and should be considered for future mechanistic studies on gut aging.

## Figures and Tables

**Table 1:** Baseline patient sample characteristics.

	Pediatric	Adult	Geriatric	*p*
**Age, Years**				
Mean ± SD	16 ± 2.1	39.5 ± 10.6	74.6 ± 8.5	*p* < 0.0001 *
**Sex**				
Male	0	4	4	
Female	5	4	3	
**BMI, kg/m^2^**				
Mean	18.8	26	25.8	*p*_PA_ = 0.021 *, *p*_PG_ = 0.016 *, *p*_AG_ = 0.920
95% CI	15.1–22.5	21.5–30.6	21.4–30.1	
**Reduction of Lymphoid Follicles**	20% (1/5)	62.5% (5/8)	85.7% (6/7)	*p*_PA_ = 0.265, *p*_PG_ = 0.072, *p*_AG_ = 0.569
**Fibrous Obliteration of Tip**	20% (1/5)	75% (6/8)	71.4% (5/7)	*p*_PA_ = 0.103, *p*_PG_ = 0.242, *p*_AG_ > 0.999

BMI—body mass index. SD—standard deviation. CI—confidence interval. P—pediatric, A—adult, G—geriatric. Data are presented as mean ± SD or 95% CI and analyzed using Student’s *t*-test or Fisher’s exact test. * *p* < 0.0001.

## Data Availability

Raw sequencing reads used in this study are available from the ENA repository under Study Accession PRJEB104020.

## Supplementary Materials

The following supporting information can be downloaded at: https://www.mdpi.com/article/10.3390/ijms262311399/s1.

## Abbreviations

The following abbreviations are used in this manuscript:

AMPA	α-Amino-3-hydroxy-5-Methyl-4-isoxazolePropionic Acid
BMI	Body Mass Index
DEG	Differentially Expressed Gene
ENS	Enteric Nervous System
FDR	False Discovery Rate
GALT	Gut-Associated Lymphoid
GO	Gene Ontology
ISCs	Intestinal Stem Cells
KEGG	Kyoto Encyclopedia of Genes and Genomes
PCA	Principal Component Analysis
ROS	Reactive Oxygen Species
TER	Transepithelial Electrical Resistance
