# Supplementary figures and images for: Age-Related Transcriptomic Changes in the Vermiform Appendix
Source: Int J Mol Sci. 2025 Nov 25;26(23):11399. doi: 10.3390/ijms262311399 (PMC12692331; doi:10.3390/ijms262311399)

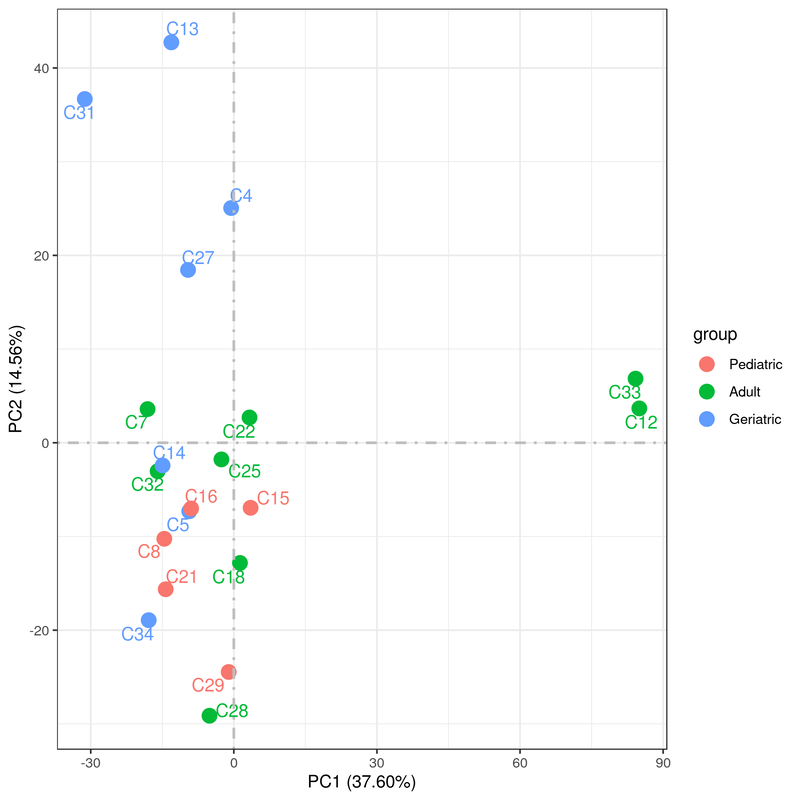

Supplement: Supplementary file 1 [file ijms-26-11399-s001.zip › Supplemental_figure_1.png]
